# Biochar Effectively Promoted Growth of *Ardisia crenata* by Affecting the Soil Physicochemical Properties

**DOI:** 10.3390/plants13131736

**Published:** 2024-06-23

**Authors:** Muqi Niu, Xiuming Chen, Yun Pan, Shunshun Wang, Luyu Xue, Yanru Duan, Sagheer Ahmad, Yuzhen Zhou, Kai Zhao, Donghui Peng

**Affiliations:** 1 Cross-Strait Floriculture Industry Science and Technology Innovation Hub, Fujian Ornamental Plant Germplasm Resources Innovation & Engineering Application Research Center, Key Laboratory of National Forestry and Grassland Administration for Orchid Conservation and Utilization, College of Landscape Architecture and Art, Fujian Agriculture and Forestry University, Fuzhou 350002, China; muqiniu@fafu.edu.cn (M.N.); chenxiuming@fafu.edu.cn (X.C.); yunpan@fafu.edu.cn (Y.P.); shunshunwang@fafu.edu.cn (S.W.); luyuxue@fafu.edu.cn (L.X.); yanruduan@fafu.edu.cn (Y.D.); sagheerhortii@gmail.com (S.A.); zhouyuzhencn@fafu.edu.cn (Y.Z.); 2 College of Life Sciences, Fujian Normal University, Fuzhou 350117, China

**Keywords:** *Ardisia crenata*, biochar, soil properties, root morphology, photosynthesis, growth

## Abstract

Biochar is regarded as a soil improvement material possessing superior physical and chemical properties that can effectively enhance plant growth. However, there exists a paucity of research examining the efficacy of biochar in supplanting traditional materials and its subsequent impact on the growth of *Ardisia crenata*, which is currently domesticated as fruit ornamentals. In this study, the mechanism of biochar’s effect on *Ardisia crenata* was analyzed by controlled experiments. For 180 days, their growth and development were meticulously assessed under different treatments through the measurement of various indices. Compared with the references, the addition of biochar led to an average increase in soil nutrient content, including a 14.1% rise in total nitrogen, a 564.1% increase in total phosphorus, and a 63.2% boost in total potassium. Furthermore, it improved the physical and chemical properties of the soil by reducing soil bulk density by 6.2%, increasing total porosity by 6.33%, and enhancing pore water by 7.35%, while decreasing aeration porosity by 1.11%. The growth and development of *Ardisia crenata* were better when the appending ratio of biochar was in the range of 30% to 50%, with the root parameters, such as root length, root surface area, and root volume, 48.90%, 62.00%, and 24.04% higher to reference. At the same time, the biomass accumulation of roots in the best group with adding biochar also increased significantly (55.80%). The addition of biochar resulted in a significant improvement in the content of chlorophyll a and chlorophyll b (1.947 mg g^−1^) and the net photosynthetic rate (5.6003 µmol m^−2^ s^−1^). This study’s findings underpinned the addition of biochar in soil improvement and plant response. Therefore, biochar can favor the cultivation and industrial application of *Ardisia crenata* in the future, leading to an efficient and environmentally friendly industrial development.

## 1. Introduction

Currently, the continuous growth of the global population has resulted in an increasing scarcity of land resources. Over-exploitation and improper use have led to a decline in soil quality, a disruption of ecological balance, an exacerbation of climate change, and increased threats from diseases and pests, among other global issues [1]. To mitigate environmental damage and enhance the sustainability of agricultural production, it is imperative to develop novel strategies that can replace current management methods [2]. In recent years, soilless cultivation has attracted global attention due to its ease of management and high cost-effectiveness [3]. Compared to traditional cultivation methods, the soilless cultivation method can conserve land resources, water, and nutrients while more precisely controlling plant growth environmental conditions for economic plant cultivation [4,5].

In recent years, biochar has become a popular cultivation medium with significant potential due to its exceptional physical and chemical properties as well as rich nutritional components [6]. It is a porous material with high C content, produced by pyrolysis at 300–700 °C through different processing techniques [7,8]. There exist a porous structure, high specific surface area, and large pore volume in this material [9]. Numerous previous studies consistently demonstrated that biochar holds promise for enhancing the physical and chemical properties of soil, such as reducing bulk density [10]; improving water retention [11]; enhancing soil nutrient utilization efficiency [12]; remediating soil pollution [13]; augmenting carbon sequestration capacity [14]; increasing the total nitrogen, total phosphorus, and total potassium contents in the soil [15,16,17]; as well as promoting enhanced cycling of nutrients within the soil environment [18]. Generally, various characteristics of biochar are affected by pyrolysis temperature, microstructure reconstruction sequence, and raw materials, resulting in great differences in plant growth and development [19,20,21,22]. For instance, the application of biochar derived from straw has been proven to enhance wheat and maize yields, improve phenotypic traits, and increase biomass accumulation [23,24]. Additionally, wood biochar has been found to positively impact radish root yield while initially showing a negative effect on the germination of certain crops [25]. At the same time, straw biochar can also improve the soil environment by enriching soil microbial communities and enhancing enzyme activity, enhancing soil nutrient utilization efficiency, and promoting the cycle of carbon, nitrogen, and phosphorus [26]. Moreover, multiple studies have shown that the addition of biochar can increase the total root length (TRL), root surface area (TPA), and root volume (RV) of plants such as maize [27,28]. The increase in nutrients and soil pores can also have an effect on the root tissue density (RTD) and root diameter (RD) of plants, thereby improving root space and promoting root development [28,29,30]. Despite the significant potential of biochar as a plant cultivation medium or soil amendment in improving soil properties and promoting crop growth and development, its application value and development for ornamental plants are comparatively limited.

*Ardisia crenata* Sims is an evergreen shade-tolerant shrub in the primula family [31]. It is an ornamental plant with bright fruits, an extremely long fruit-bearing period, and a strong particulate matter (PM) reduction and adsorption effect [32,33]. It also has high medicinal value. Lactones extracted from its leaves have great antibacterial and anti-inflammatory activities [34]. Studies on *A. crenata* showed that it contained a variety of saponins or medicinal components showing good healing ability for a variety of diseases [35]. The roots of *A. crenata* are widely used in traditional Chinese medicine for respiratory tract infections [36]. Due to its high ornamental and medicinal value, it is widely cultivated in South China’s Fujian Province, while the varieties used for ornamental purposes are also popular in Southeast Asian countries such as Vietnam. Despite widespread cultivation of *A. crenata* in China, there remains a lack of research on its cultivation practices, including the scientific nature of the commonly used mixed matrix of peat and coconut shell. Furthermore, there are limited reports on the cultivation of emerging materials such as biochar, which hinders both the ornamental quality and industrial development of *A. crenata*.

This experiment aims to investigate the effects of different biochar addition ratios within mixed matrices on the phenotypic characteristics, root morphology, and physiological traits of *A. crenata* while simultaneously evaluating biochar’s potential as a substitute for traditional culture medium based on plant growth conditions.

## 2. Materials and Methods

### 2.1. Preparation of Cultivation Medium

The biochar utilized in this experiment was obtained by an agricultural company located in Pingdingshan, China. The primary raw materials consisted of wheat straw and a small amount of wood chips. The raw materials were cleaned, dried, and pulverized and then placed in a tube furnace for anaerobic pyrolysis at a temperature of 700 °C and a reaction time of 2 h. Subsequently, they were allowed to cool before being extracted and sieved.

Both the peat and coconut husk were sourced from local agricultural companies, and before the experiment began, all the culture substrates were sun-sterilized and dried to facilitate various subsequent performance measurements. The solarization process used a single layer of plastic film to cover the cultivation substrate laid on a flat surface and was carried out for one week on a sunny summer day [37].

### 2.2. Experimental Treatments

The experiment was conducted in the greenhouse of Fujian Agriculture and Forestry University in Fujian Province, China. *A. crenata* plants were grown in pots with a container size of 18 × 16 cm, one plant per pot. The control group was treated with a medium of an equal volume ratio of peat to coconut husk (commonly used in *A. crenata* production). On the basis of the control group, biochar of 20%, 30%, 40%, 50%, 60%, and 100% was added to the different treatment groups, the different treatment groups were labeled as BC (0~100) according to the volume ratio of biochar, and 30 pots were planted in each treatment. *A. crenata* were planted in June 2023. The growth and physiological characteristics of the plants were measured in October 2023 (90 ds). The growth and physiological characteristics were measured in January 2024 (180 ds). In order to ensure that the experimental results were not affected, other management measures were kept consistent during the experiment except for adding different volumes of biochar.

### 2.3. Analysis of the Properties of Cultivation Medium

The cutting rings method was used to measure the physical properties of each treatment under different biochar volume ratios, including bulk density, total porosity, ventilation porosity, water-holding porosity, and air–water ratio [38,39,40]. A ring knife with a volume of 100 cm^3^ was taken and weighed as W_1_. The matrix was evenly mixed in different proportions and then placed into the ring knife and weighed as W_2_ after drying. The ring knife was flipped upside down and immersed in water, soaked for 24 h, and then removed and weighed as W3. The ring knife was inverted to allow for the water to drain naturally and then weighed as W4. Finally, the data were input into the formula for calculation:

Bulk density (BD) (g cm^−3^): (W_2_ − W_1_)/100

Total porosity (TPO): (W_3_ − W_2_) × 100%

Ventilation pores (VP): (W_3_ − W_4_) × 100%

Water-holding pores (WHP): Total porosity (TPO) − Ventilation pores (VP)

Air-to-water ratio (AWR): Water-holding pores (WHP)/Ventilation pores (VP)

The chemical properties of the matrix under different biochar volume ratios were measured, including pH; electrical conductivity (EC); and the contents of three elements, total nitrogen (TN), total phosphorus (TP), and total potassium (TK); as well as the cation exchange capacity (CEC). Different matrices were soaked in distilled water for 24 h, respectively, and the suspension was used to measure the pH value and electrical conductivity (EC) of the sample [41].

The cation exchange capacity (CEC) was determined by the EDTA–ammonium salt method [39,42,43]. A 1.00 g soil sample was sieved and placed into a 100 mL centrifuge tube. Next, 5 mL of an EDTA–ammonium acetate mixture was added along the tube wall and stirred until the entire sample reached a uniform mud state. Additional EDTA–ammonium acetate mixture was added to reach a total volume of 80 mL, stirred for 1–2 min, and then put into a centrifuge at a speed of 3000 RPM for 5 min; the supernatant was removed, and 95% alcohol without ammonium ions was added and stirred until the reaction had no ammonium ions. Then, a small amount of tap water was added into the tube, and the solution was stirred into a paste with a glass rod and washed into a 150 mL Kelvite bottle; the volume of washing was controlled at approximately 100 mL. A total of 2 mL liquid paraffin and 1 g magnesium oxide were added. Then, the distillation was carried out in a nitrogen analyzer, and the blank test was carried out. The result was then calculated.

The content of total nitrogen in the soil samples was determined by the semi-micro Kjeldahl method [44,45]; the soil sample was weighed, and 1.00 g was placed into a conical bottle, followed by the addition of 2 mL of catalyst (K_2_SO_4_:CuSO_4_:Se = 100:10:1) and 5 mL of concentrated sulfuric acid. After thorough shaking, the bottle was heated on an electric heating plate at 150 °C for 10 min, then at 350 °C for another 10 min, and finally at 420 °C for 60 min until a pale, clear solution was obtained. Subsequently, the solution was allowed to cool before conducting a distillation assay.

The content of total potassium (TK) was determined by flame spectrophotometry [46,47]. The sieved, air-dried soil sample was weighed, 0.4 g was transferred into a crucible, 5 mL of nitric acid was added, 5 mL of hydrofluoric acid was added, and 5 mL of perchloric acid was added, and the crucible was placed on an electric heating plate set at 180 °C. The sample was dissolved until nearly dry, and then it was cooled; a total of 4 mL of hydrochloric acid was added for 5 min. Finally, the volume was adjusted to 50 mL with distilled water before measuring using a flame spectrophotometer with the default settings.

The content of total phosphorus (TP) was determined by molybdenum antimony colorimetry [48]. Firstly, 0.500 g of air-dried soil sample was placed into a conical bottle, and 5 mL of distilled water was added. Then, 5 mL of concentrated sulfuric acid and 4 mL of perchloric acid were introduced. Subsequently, the bottle was placed on an electric heating plate for digestion and allowed to cool upon completion. The solution was transferred from the tube to a 100 mL volumetric bottle and diluted with distilled water to the mark; a total of 10 mL of the test solution was transferred to a 50 mL colorimetric tube filled with water up to 40 mL. Then, a drop of 2,4-dinitrophenol indicator was added. The pH of the solution was adjusted to a slight yellow color using a 0.5 mol/L sulfuric acid solution and a 2 mol/L sodium hydroxide solution. Next, 5 mL of molybdenum-antimony inhibitor was introduced, the volume was adjusted to 50 mL with water, and it was mixed well before performing the colorimetric measurements at a wavelength of 880 nm after an interval of thirty minutes.

To reduce errors, three repeated measurements were performed on different indicators.

### 2.4. Measurement of Plant Growth Parameter

The plant height (PH) and ground diameter (GD) of the plants under different treatments were measured at the beginning of the experiment (June 2023), 90 ds later (October 2023), and 180 ds later (January 2024). After the experiment, 6 plants from each treatment were randomly selected. The roots were cleaned with distilled water, and excess water was removed with absorbent paper. The fresh weight of the plant was recorded as the root fresh weight (RFW), stem fresh weight (SFW), and leaf fresh weight (LFW). Then, the plants were placed in an oven at 105 °C for 30 min, followed by drying at 65 °C until the weight no longer changed [49,50]. After completion, it was recorded as leaf dry weight (LDW), stem dry weight (SDW), and root dry weight (RDW).

### 2.5. Measurement of Plant Physiological Parameters

The leaves of the plants were collected at the end of the experiment for the measurement of physiological indicators. To measure the content of chlorophyll and carotenoids, the ethanol extraction method was used [51]. The collected leaves were cut into pieces and soaked in ethanol with a concentration of 95% for 48 h under dark conditions. The absorbance of the solution was measured at wavelengths of 665 nm, 649 nm and 470 nm, its content was calculated.

The soluble sugar content was determined using the anthraquinone colorimetric method [52]. A 0.10 g leaf sample was weighed and homogenized with 1 mL of distilled water and then transferred to a centrifuge tube and placed in a boiling water bath for 10 min before being centrifuged at 8000 RPM for another 10 min after cooling. The supernatant was then transferred to a large test tube, and the volume was adjusted to 10 mL with distilled water. Next, 40 μL of the sample liquid was mixed with 40 μL of distilled water along with 200 μL of concentrated sulfuric acid and 40 μL of anthranone working agent. This mixture was then immersed in a water bath at 95 °C for another 10 min, and then it was cooled; an appropriate amount was transferred to a cuvette for measurement of the OD value at a wavelength of 620 nm.

The method used for measuring soluble protein was the bicinchoninic acid assay (BCA) [53]. A total of 1 mL of buffer was added to 0.10 g of sample and shook well. Then, it was centrifuged at 10,000 RPM in a low-temperature centrifuge for 10 min after placing the sample in an ice bath. After completion, the supernatant was collected. Next, BCA working solution was added to the supernatant and incubated at 60 °C for 30 min. Finally, the OD value at 562 nm was determined.

### 2.6. Measurement of Plant Photosynthesis

After the experiment, the Li-6800 XT portable photosynthesis device (Li-COR, Lincoln, NE, USA) was used to measure the plant’s net photosynthetic rate (Pn), transpiration rate (Tr), intercellular CO_2_ concentration (Ci), and stomatal conductance (Gsw). From 9 a.m. to 11 a.m. on a sunny day, we measured the third to fifth mature functional leaves from the top of the plant under each treatment.

### 2.7. Measurement of Root Parameters

Distilled water was used to clean the plant roots, which were then placed on a transparent tray. Distilled water was poured to spread the plant roots evenly and avoid overlapping. Plant root samples were scanned using a plant root scanner (HM-GX02, Meiheng China, Inc., Weifang, China) at a speed of 300 points per inch. The root system image obtained was analyzed using WinRHIZO Pro root analysis software (version 2017a; Québec City, QC, Canada) to obtain the total root length (TRL), total projected area (TPA), total surface area (TSA), average root diameter (AD), and root volume (RV). The number of root tips (RT), root forks (RF), and root crossings (RC) were also obtained. At the same time, the specific root length (SRL), specific root surface area (SRA), and root tissue density (RTD) of the plants were also calculated.

### 2.8. Statistical Analysis

All data were analyzed using IBM SPSS 19.0 (SPSS Inc., New York, NY, USA) software for one-way analysis of variance and Duncan’s multiple range test, and it was assumed that there was a significant difference at *p* ≤ 0.05. At the same time, Pearson correlation analysis was conducted on all traits to assess the correlation between plant and soil traits. The membership function method was then used to comprehensively evaluate the various trait parameters under different treatments. Then, Origin 2022 (OriginLabInc, Northampton, MA, USA) software was used to further plot the data and its conclusions.

Membership function value R(X_i_): (X_i_ − X_min_) − (X_max_ − X_min_)

Inverse membership function value R(X_i_): 1 − {(X_i_ − X_min_) − (X_max_ − X_min_)}

In the formula, X_i_ represents the measured value of a certain measurement index, and X_max_ and X_min_ represent the maximum and minimum values of the measured values.

## 3. Results

### 3.1. Soil Physical and Chemical Properties and Nutrient Content

The main focus was on measuring the changes in physical and chemical properties as well as the key nutrients across different treatment groups. The pH of the biochar was 8.67, whereas the pH of the control group (PC) was 5.30. Compared to the treatment group without biochar addition, the addition of biochar resulted in an increase in cation exchange capacity (CEC) and higher levels of total phosphorus, total potassium, and total nitrogen. However, there was no change in conductivity values (Table 1). Obviously, compared with PC (BC0), biochar has a smaller volume density and lighter weight (Table 2). In terms of water-holding capacity, biochar has larger total porosity and water-holding pores, but the ventilation pores appear to be inferior to PC (Table 2). By continuously increasing the mixed matrix, the volume ratio, total volume density, ventilation holes, and air-to-water ratio of biochar continued to decrease, while the total porosity and water-retaining pores continued to increase, indicating the different physical properties of biochar and the peat coir matrix (PC).

### 3.2. Growth Parameters of A. crenata

**Figure 1:**
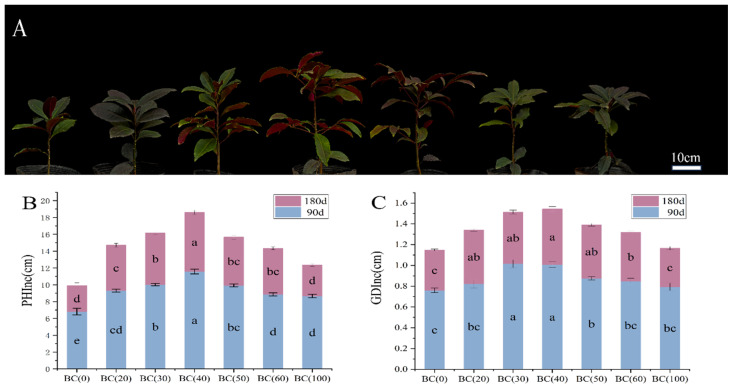
Effect of biochar on the aboveground phenotype of *A. crenata* under different treatments. (**A**) Typical photos of shoot growth and development of *A. crenata* under different treatments. From left to right in turn for BC (0), BC (20), BC (30), BC (40), BC (50), BC (60), and BC (100). (**B**) Effects of different biochar treatments on plant height growth. (**C**) Effects of different biochar treatments on ground diameter growth. Different colors indicate the amount of growth in different seasons. The significance of differences was determined by one-way analysis of variance (ANOVA) using Duncan’s multiple range test. Different letters indicate significant differences at *p* ≤ 0.05.

The results presented in Figure 1A demonstrate significant variations in the growth of *A. crenata*, specifically in terms of plant height and ground diameter, across two distinct growing seasons. In comparison to the treatment without biochar addition (BC0), the inclusion of biochar resulted in enhanced plant height. Notably, a trend of initial increase followed by decrease was observed with increasing amounts of biochar, reaching its maximum at 40% addition (BC40) (Figure 1B). Similarly, the growth of ground diameter was significantly influenced by biochar; however, excessive additions had negative effects (Figure 1C). Furthermore, when comparing the two growing seasons, a low increasing rate was observed during the second season due to seasonal effects.

**Figure 2:**
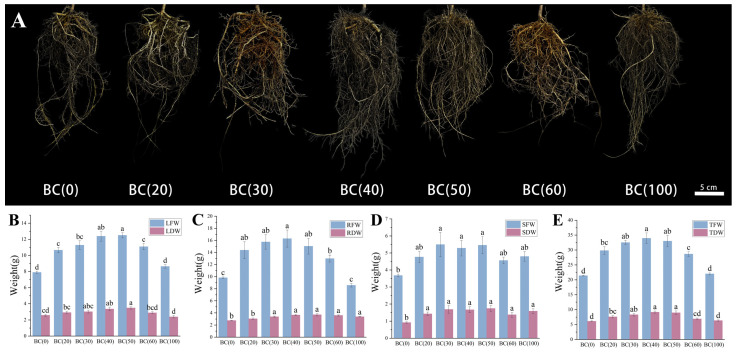
Effects of different biochar treatments on the underground site and biomass of *A. crenata*. (**A**) Typical photos of roots under different biochar treatments (Photo was taken in January 2024). (**B**) Changes in fresh and dry weight of leaves under different biochar treatments. (**C**) Changes in fresh weight and dry weight of roots under different biochar treatments. (**D**) Changes in fresh weight and dry weight of stems under different biochar treatments. (**E**) Changes in overall fresh weight and dry weight of plants under different biochar treatments. Values are shown as mean ± standard error (n = 6). The significance of differences was determined by one-way analysis of variance (ANOVA) using Duncan’s multiple range test. Different letters indicated significant differences at *p* ≤ 0.05.

Compared to the treatment without biochar addition, the inclusion of biochar significantly influenced the biomass of both the upper and lower sections of the *A. crenata* plants. When the biochar addition ratio is between 20% and 60%, the biochar matrix has a significant positive impact on the LFW, RFW, and TFW of *A. crenata* (Figure 2B,C,E).

It was worth noting that the trend in SFW appeared to follow a regular pattern, regardless of the changes in the proportion of biochar, with overall growth being better than that observed in the treatment without biochar addition (Figure 2D). When the biochar addition reached 40% (BC40), the LFW, RFW, and TFW all reached their maximum values. This indicated that the addition of biochar possibly had a positive impact on the growth and development of both the upper and lower parts of *A. crenata* by increasing soil pH, enhancing CEC, and raising the levels of key soil nutrients. However, when the addition exceeded 40%, a decrease in plant performance was observed, suggesting that excessive biochar application may have resulted in detrimental effects. This could be attributed to issues related to drainage and aeration due to an excessively high proportion of water-holding pores and inadequate aeration pores as well as potential nutrient imbalances caused by the elevated biochar ratio.

### 3.3. Root Parameters of A. crenata

Compared with the treatment without the addition of biochar, the *A. crenata* with the addition of biochar had better root development, such as more fibrous roots and larger root volume (Figure 2A). Compared with the non-biochar treatment, TRL, TPA, TSA, and RV were increased in the biochar treatment, while AD was decreased, but it was not significant. The addition of biochar could promote the development and growth of *A. crenata* root (Table 3). Similarly, RT, RF, and RC also increased significantly compared to the treatment without biochar addition. SRL and SRA showed an increasing trend, while RTD showed a decreasing trend (Table 4).

### 3.4. Plant Physiological Parameters

**Figure 3:**
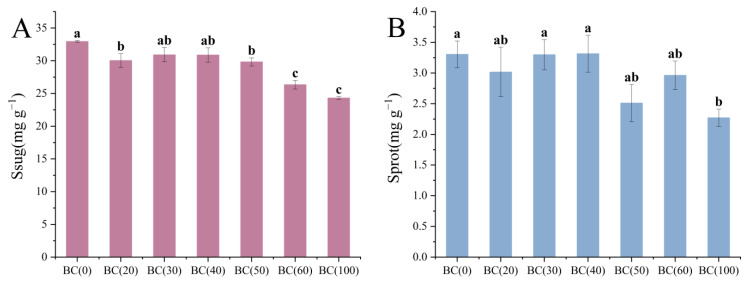
Effects of different treatments on osmotic regulating substances in *A. crenata.* (**A**) The effect of biochar on soluble sugar. (**B**) The effect of biochar on soluble protein. Values are shown as mean ± standard error (n = 3). The significance of differences was determined by one-way analysis of variance (ANOVA) using Duncan’s multiple range test. Different letters indicate significant differences at *p* ≤ 0.05.

**Figure 4:**
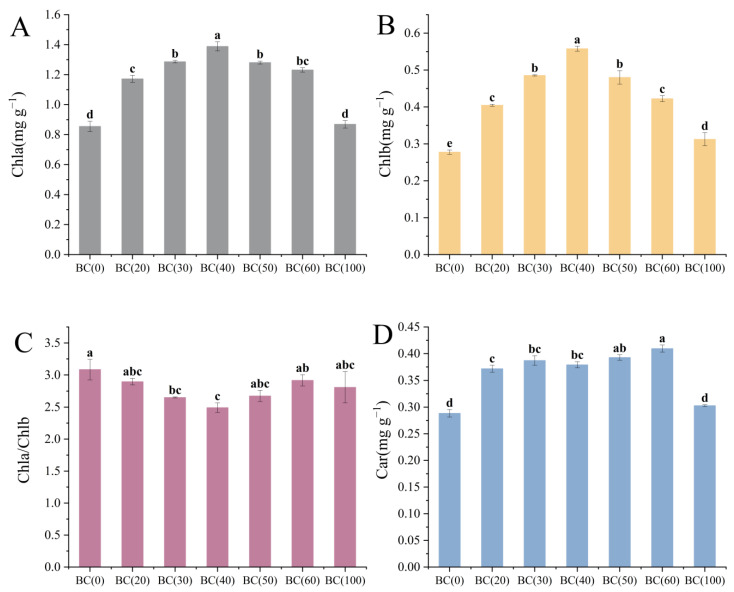
Effects of different treatments on chlorophyll and carotenoid content in *A. crenata*. (**A**) (chlorophyll a). (**B**) (chlorophyll b). (**C**) (chlorophyll a/chlorophyll b). (**D**) (carotenoids). Values are shown as mean ± standard error (n = 3). The significance of differences was determined by one-way analysis of variance (ANOVA) using Duncan’s multiple range test. Different letters indicate significant differences at *p* ≤ 0.05.

Soluble sugar was decreased with the increase in the biochar addition ratio (Figure 3A). However, the soluble protein content was not changed significantly (Figure 3B). The decrease in soluble sugar might be caused by the consumption of biochar addition, which improved the utilization efficiency of the plants during the vigorous growth period in summer, but it might also be caused by the drainage and exhaust problems caused by excessive biochar addition in high-temperature weather. At the same time, the addition of biochar also affected the photosynthesis of the plants (Figure 4). Compared with the treatment group without biochar addition, the addition of 20–40% (BC20–40) biochar significantly affected the Chla and Chlb (Figure 4A,B), reaching maximum content at 40% biochar. Car is also affected by the addition of biochar, but the impact of the addition ratio is not significant (Figure 4D). It is worth noting that the ratio of Chla to Chlb first decreased and then increased as the biochar addition ratio increased (Figure 4C), and the ratio was the smallest when the addition ratio was 40% (BC40). Given that *A. crenata* is a shade-loving plant, this change may have enhanced its photosynthetic efficiency in low-light environments.

### 3.5. Plant Photosynthesis Parameters

**Figure 5:**
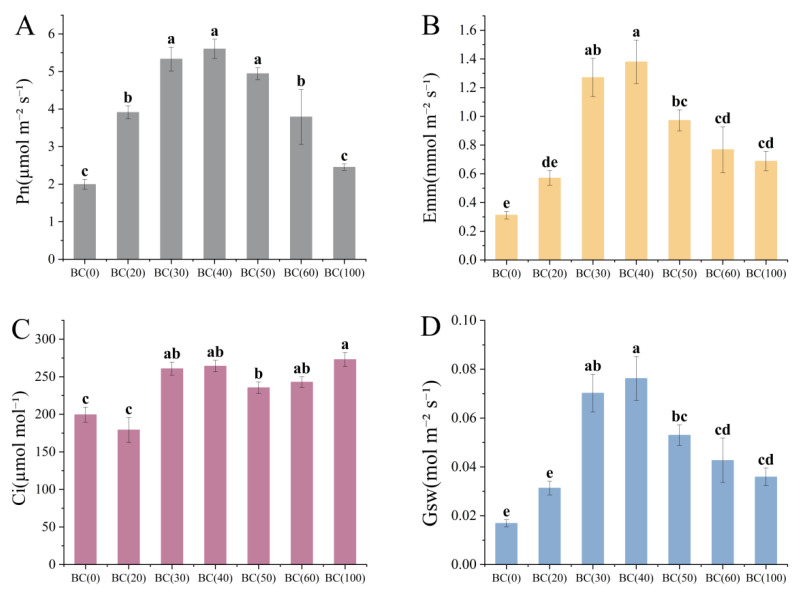
Effects of different treatments on photosynthesis of *Ardisia crenata*. (**A**) (Net photosynthetic rate). (**B**) (Transpiration rate). (**C**) (Intercellular CO_2_ concentration). (**D**) (Stomatal conductance). Values are shown as mean ± standard error (n = 3). The significance of differences was determined by one-way analysis of variance (ANOVA) using Duncan’s multiple range test. Different letters indicate significant differences at *p* ≤ 0.05.

The results showed that the treatment with biochar showed higher Pn, Tr, and Gsw compared with the treatment without biochar addition (Figure 5). However, their concentration was decreased at 40% biochar.

Unlike the other indicators, Ci was positively affected by the addition of biochar (Figure 5C).

### 3.6. Correlation Analysis between Plant Traits and Soil Matrix Properties

**Figure 6:**
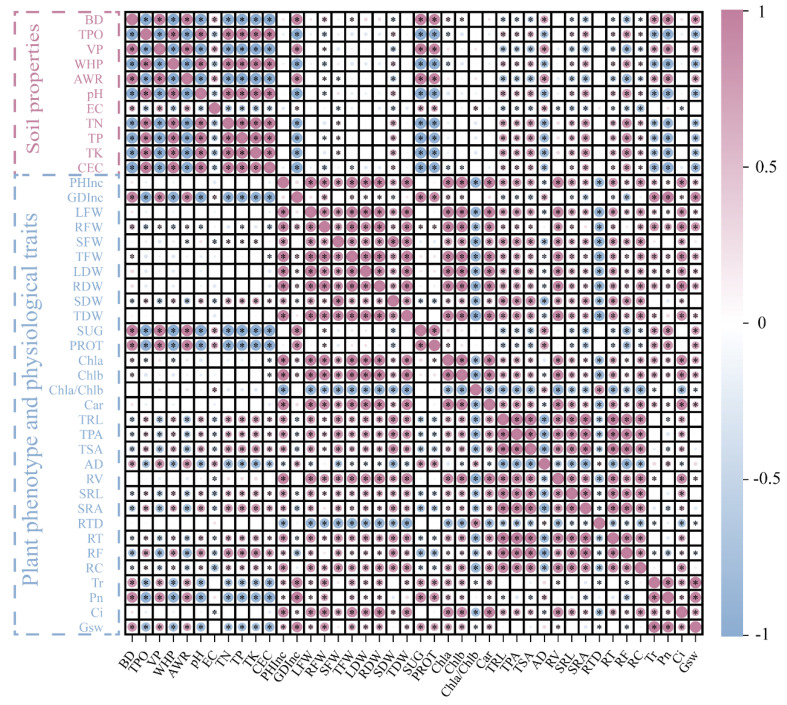
Pearson correlation heat map between plant traits and soil traits. The size and color of the circle in the figure indicate the size of the R^2^ value; * indicates that the level is significant when *p* < 0.05. Increases and decreases in abundance are shown in purple and blue, respectively.

In the relationship between plants and soil, BD, VP, and AWR were positively correlated with PHInc, GDInc, and LFW in the aboveground parts of the plants but negatively correlated with the underground parts. TN, TP, and TK had no significant effect on the aboveground parts of the root system but had a greater effect on the underground parts of the root system. It was worth noting that pH was negatively correlated with aboveground sites but positively correlated with underground sites (Figure 6).

**Figure 7:**
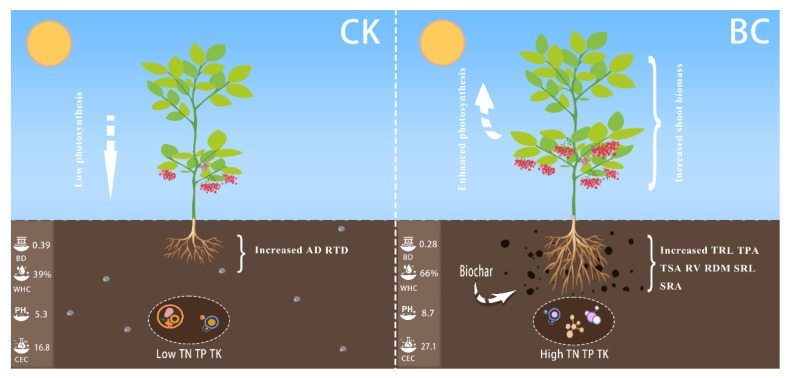
Summary diagram of effects of biochar treatment on soil and *A. crenata*. CK represents a treatment without biochar addition, and BC represents a treatment with biochar addition.

Soluble sugar (SUG) and soluble protein (PROT) were positively correlated with BD, VP, AWR, and EC but negatively correlated with TPO, WHP, pH, TN, TP, and TK. A comparison of photosynthetic pigments and photosynthetic parameters found that Chla, Chlb, and Chla/Chlb were positively correlated with BD, VP, and AWR and negatively correlated with TPO, WHP, pH, TN, TK, TP, and CEC; in addition, Chlb was negatively correlated with EC, and Chla was negatively correlated with Chla/Chlb. In terms of the photosynthetic parameters, Tr, Pn, and Gsw showed positive correlations with BD, VP, pH, TN, TP, and TK but negative correlations with TPO, WHP, AWR, and EC. The relationship between plants and soil revealed that the incorporation of biochar had a positive impact on the physical and chemical properties of the soil, resulting in an improved soil environment conducive to the growth of *A. crenata* plants. Additionally, these enhancements in soil properties contributed to the growth, development, and photosynthetic activity of the aboveground parts of the *A. crenata* plants (Figure 7).

### 3.7. Comprehensive Evaluation of Plant Growth Physiological Indexes

The membership function was used to evaluate the indexes of *A. crenata*. The higher the mean value, the better the plant growth under treatment (Table 5). In terms of the growth and physiological characteristics of each plant, BC (40) treatment performed the best, followed by BC (50) and BC (30) treatments. The comprehensive evaluation of BC (40) is only 0.021 points with BC (50) and 0.076 points with BC (30), but the comprehensive evaluation of BC (20), BC (60), BC (0), and BC (100) is significantly different. In conclusion, adding the proper proportion of biochar to the substrate is beneficial to plant growth.

## 4. Discussions

### 4.1. The Plant Growth Promotion Effect of Biochar by Altering the Physiochemical Properties of Soil

As a soil conditioner with high sustainability and a wide range of sources, biochar’s positive effects on plant growth and development through the improvement of the physical and chemical properties of soil have been widely confirmed in numerous studies [54,55]. On the physical level, biochar interacts and aggregates with other mineral particles in soil through the particularity of its porous material, reducing soil bulk density (BD); studies have also shown that biochar can reduce particle density and increase soil porosity and water holding capacity by improving the stability of wet aggregates [56], provide friendly support for plants to improve water utilization capacity and increase available water amount [57], and provide certain support for the construction of microbial communities due to its good physical structure [10]. However, excessive biochar addition may have a negative impact on plants. In the present study, when the ratio of biochar addition exceeds 40%, the growth and development of the plants are limited to a certain extent, which may be caused by too light bulk density, too large soil pores, and poor exhaust gas, which cannot provide good support for plant root growth [58]. In addition to the aforementioned improvements in soil physical properties facilitated by the biochar application mentioned above, research also suggests that biochar significantly impacts parameters such as the soil penetration resistance, tensile strength, and shear strength coefficient of linear extensibility, leading to some alterations in cracking behavior [56].

Biochar typically exhibits alkaline properties, thereby leading to an increase in soil pH value. Additionally, its rich mineral ion content can elevate the cation exchange capacity (CEC) [59,60,61]. Research has demonstrated that biochar addition may impact NH_4_^+^ storage in soil by enhancing the cation exchange capacity [62]. Moreover, nitrifying bacteria and adsorption of ammonium salt in soil are further affected to affect the N cycle in soil [63,64,65,66], and these processes also contribute to reducing soil salt leaching and nutrient loss [67,68]. At the same time, the high specific surface of biochar can also increase the utilization efficiency of soil phosphorus through adsorption, and biochar derived from manure or straw can also provide more organic or inorganic P [18]. In other studies, it was found that the addition of biochar can promote the activity of potassium-soluble bacteria in soil [69], thus promoting the dissolution of potassium-containing substances in soil, and the addition of biochar could also increase the amount of potassium absorbed by various parts and organs of plants [70]. In our study, biochar was found to increase the content of nitrogen, phosphorus, and potassium in the soil (Table 1), thereby facilitating nutrient cycling and enhancing the availability of effective nutrients in the soil. It is important to note that the effects of biochar on soil modification are time-dependent and can vary based on different materials. Research has indicated that biochar undergoes aging over time due to its interaction with the soil [71], leading to alterations in micro-structure and function [72]. In the long-term, reapplication of biochar at regular intervals or in combination with suitable fertilizers proves more advantageous for plant growth, as evidenced by longer-term experiments [73,74].

### 4.2. Effects of Biochar Addition on Plant Roots

The root system, in direct contact with the soil environment, plays a crucial and irreplaceable role in the absorption, uptake, and transformation of essential plant nutrients. Extensive prior research has unequivocally demonstrated that integrating biochar into soil ecosystems can profoundly enhance various aspects of plant roots, such as increasing the root length, root surface area, and root volume of plant roots as well as increasing the number of root tips, branches, and nodules of legumes [28,30,39]. The reasons for these advantages are varied, for example, the porous nature of biochar increases soil porosity and reduces bulk density, which removes more restrictions on plant root growth [58], or biochar enhances soil nitrogen, phosphorus, and other nutrient cycling [15]; additionally, research indicates that incorporating biochar can offer improved support for promoting the healthy growth of plant roots even in polluted or contaminated soils [75].

In this study, the root biomass (RDW), total root length (TRL), total surface area (TSA), and volume of plant roots (RV) increased with the increase in the proportion of biochar added, which may be due to the promotion effect caused by the increase in nutrients in the soil (Table 1) [76]. Our findings suggest that the increased availability of essential nutrients, such as nitrogen, phosphorus, and potassium, may have triggered the growth and development of plant roots. However, when the proportion of biochar added exceeded 40%, the performance of root growth began to decline. This may be because the alkaline pH caused by the high proportion of biochar addition is unfavorable to the root system, even though studies have shown that the increase in pH of biochar is conducive to the growth of plant roots; the appropriate pH of different plants should be considered separately [27]. Meanwhile, the increase in ventilation pores and water-holding pores will also exert certain pressure on the root system.

The alterations in root parameters often serve as a direct indicator of soil modifications, offering valuable insights into the effects of soil amendments such as biochar. Plants planted in soils with more abundant soil resources usually exhibit larger specific root length and specific surface area [77], while plants planted in soils with poor or bad properties usually have higher average root diameter and higher root tissue density [78]. In this study, the average diameter (AD) of the plant roots and the density of root tissues (RTD) did not change significantly, which may be caused by little change in the availability or storage of nutrients in the soil under the influence of biochar. Although the total nitrogen and total phosphorus in the soil have been increasing with the increase in the proportion of biochar added, it is uncertain whether the ratio of available nitrogen to available phosphorus has increased significantly.

### 4.3. Effects of Biochar on Shoot Parts and Photosynthesis of Plants

The effects of biochar addition on the aboveground parts of plants were usually indirect, as the two are not in direct contact. The development strategy of plants was usually determined by the efficiency of nutrient use [79]. Studies have shown that biochar addition improves crop productivity by enhancing nitrogen and phosphorus absorption, thereby indirectly improving aboveground traits [80,81]. For example, the improved soil flora after the addition of biochar will improve the quality and yield of some cash crops, such as soybeans [82], citrus [83], corn [84], etc. At the same time, under the influence of biochar addition, photosynthesis will also change.

Photosynthesis is one of the most important driving processes of plant accumulation of nutrients and growth and development [85]. Studies have shown that biochar addition can improve plant gas-exchange parameters, thereby increasing the biomass accumulation of tomato and maize plants [86,87]. The addition of 10 t ha^−1^ biochar could significantly improve the chlorophyll fluorescence of peanuts and enhance their net photosynthetic rate, transpiration rate, and stomatal conductance compared to no addition [88]. In this study, the photosynthesis of *A. crenata* was gradually enhanced when the ratio of biochar was increased, but the photosynthesis was limited when the ratio exceeded 40%, which might be due to the accumulation and imbalance of soil nutrients caused by excessive biochar addition [89]; in addition, a high C/N ratio could lead to nitrogen immobilization [18]. The improvement effect of biochar addition on photosynthesis was mainly reflected in the enhancement of microbial and enzyme activities in soil [90], thus promoting the utilization efficiency of soil nitrogen and phosphorus by plants and the application of fertilizer, thus increasing the photosynthetic pigment content of plant leaves and enhancing the photosynthetic characteristics of leaves [91]. Under the positive influence of photosynthesis, plants accumulated more compounds, thus promoting rapid growth in both underground and aboveground sites.

## 5. Conclusions

The present study investigated the impact of incorporating biochar integration into potted *A. crenata* on both plant growth and physiological indices. The addition of biochar significantly modified physical properties, such as bulk density and total porosity, thereby creating more favorable conditions for root development. Furthermore, the increase in photosynthetic pigments and the enhancement of photosynthesis also promoted the growth and development of plants to a certain extent, providing additional support for the normal growth of plants (Figure 7). The results showed that moderate biochar addition would have a positive impact on plant growth, highlighting the potential prospects of using biochar as a soil improvement matrix to enhance plant growth and development.

## Figures and Tables

**Table 1:** Chemical properties of different treatment groups.

Factor	pH	EC (ms cm^−1^)	TN (g kg^−1^)	TP (g kg^−1^)	TK (g kg^−1^)	CEC (cmol kg^−1^)
BC (0)	5.31 ± 0.012 g	2.04 ± 0.044 a	2.998 ± 0.113 d	0.941 ± 0.034 g	4.547 ± 0.263 g	16.869 ± 0.13 f
BC (20)	5.523 ± 0.02 f	1.98 ± 0.021 a	3.303 ± 0.08 c	2.86 ± 0.046 f	5.964 ± 0.041 f	16.879 ± 0.102 f
BC (30)	5.933 ± 0.046 e	1.99 ± 0.012 a	3.379 ± 0.018 bc	4.688 ± 0.013 e	6.89 ± 0.015 e	17.603 ± 0.132 e
BC (40)	6.267 ± 0.02 d	2.013 ± 0.023 a	3.393 ± 0.011 bc	6.71 ± 0.024 d	7.495 ± 0.005 d	18.551 ± 0.07 d
BC (50)	6.707 ± 0.009 c	2.01 ± 0.012 a	3.508 ± 0.041 b	8.023 ± 0.054 c	8.005 ± 0.064 c	19.701 ± 0.102 c
BC (60)	7.077 ± 0.02 b	2.03 ± 0.01 a	3.51 ± 0.007 b	8.966 ± 0.078 b	8.767 ± 0.04 b	21.179 ± 0.113 b
BC (100)	8.667 ± 0.057 a	1.97 ± 0.015 a	4.015 ± 0.04 a	15.079 ± 0.158 a	11.72 ± 0.306 a	27.163 ± 0.148 a

Notes: The values in the table are mean ± standard error (n = 3), EC (electrical conductivity), TN (total nitrogen), TP (total phosphorus), TK (total potassium), CEC (cation exchange capacity). Different letters after the values indicate significant differences at the *p* < 0.05 level in Duncan’s test.

**Table 2:** Physical properties of different treatment groups.

Factor	BD (g cm^−3^)	TPO (%)	VP (%)	WHP (%)	AWR (%)
BC (0)	0.387 ± 0.003 a	50.19% ± 0.13% e	11.64% ± 0.39% a	38.55% ± 0.52% e	0.302 ± 0.014 a
BC (20)	0.372 ± 0.002 b	53.85% ± 0.72% d	11.04% ± 0.57% ab	42.81% ± 1.28% d	0.259 ± 0.021 b
BC (30)	0.368 ± 0.002 b	54.53% ± 0.14% d	10.88% ± 0.18% ab	43.65% ± 0.21% d	0.249 ± 0.005 b
BC (40)	0.368 ± 0.003 b	53.42% ± 0.53% d	11.21% ± 0.34% ab	42.21% ± 0.79% d	0.266 ± 0.013 b
BC (50)	0.363 ± 0.004 b	58.71% ± 0.19% c	10.20% ± 0.10% bc	48.51% ± 0.29% c	0.21 ± 0.003 c
BC (60)	0.344 ± 0.003 c	62.12% ± 0.12% b	9.82% ± 0.27% cd	52.30% ± 0.08% b	0.188 ± 0.006 c
BC (100)	0.285 ± 0.003 d	74.86% ± 1.29% a	9.00% ± 0.23% d	65.86% ± 1.48% a	0.137 ± 0.006 d

Notes: The values in the table are mean ± standard error (n = 3), BD (bulk density), TPO (total porosity), VP (ventilation pores), WHP (water-holding pores), AWR (air-to-water ratio). Different letters after the values indicate significant differences at the *p* < 0.05 level in Duncan’s test.

**Table 3:** Changes in basic root configuration of *A. crenata* under different treatments.

Factor	TRL (cm)	TPA (cm^2^)	TSA (cm^2^)	AD (mm)	RV (cm^3^)
BC (0)	610.878 ± 7.8 d	33.499 ± 0.808 d	41.079 ± 0.809 d	0.548 ± 0.011	3.951 ± 0.193 d
BC (20)	762.032 ± 20.977 c	41.527 ± 0.487 c	49.819 ± 0.551 c	0.544 ± 0.02	5.657 ± 0.252 bc
BC (30)	838.686 ± 13.192 b	45.936 ± 1.277 b	58.015 ± 1.731 b	0.522 ± 0.003	6.851 ± 0.495 a
BC (40)	909.625 ± 10.536 a	53.171 ± 1.372 a	66.549 ± 1.48 a	0.521 ± 0.003	7.34 ± 0.247 a
BC (50)	936.336 ± 18.439 a	54.085 ± 1.499 a	64.537 ± 2.311 a	0.529 ± 0.004	7.56 ± 0.435 a
BC (60)	910.123 ± 6.33 a	52.691 ± 1.325 a	63.611 ± 0.856 a	0.522 ± 0.01	6.555 ± 0.249 ab
BC (100)	826.728 ± 5.672 b	45.029 ± 0.538 b	59.187 ± 0.502 b	0.517 ± 0.009	5.358 ± 0.321 c

Note: Total root length (TRL), total projected area (TPA), total surface area (TSA), average root diameter (AD), root volume (RV). Values are shown as mean ± standard error (n = 3). The significance of differences was determined by one-way analysis of variance (ANOVA) using Duncan’s multiple range test. Different letters indicate significant differences at *p* ≤ 0.05.

**Table 4:** Changes in root parameters of *A. crenata* under different treatments.

Factor	RT (k plant^−1^)	RF (k plant^−1^)	RC (k plant^−1^)	SRL (cm g^−1^)	SRA (cm^2^ g^−1^)	RTD (g cm^−3^)
BC (0)	7.767 ± 0.096 d	8.997 ± 0.057 e	1.602 ± 0.01 c	221.926 ± 2.553 b	14.928 ± 0.326 c	1.524 ± 0.042
BC (20)	8.684 ± 0.121 c	10.315 ± 0.145 d	1.945 ± 0.037 a	250.92 ± 5.442 a	16.452 ± 0.523 b	1.448 ± 0.046
BC (30)	9.063 ± 0.053 c	10.852 ± 0.184 c	1.88 ± 0.009 b	248.188 ± 4.792 a	17.154 ± 0.441 ab	1.458 ± 0.06
BC (40)	10.311 ± 0.179 a	11.888 ± 0.144 b	1.894 ± 0.007 ab	250.165 ± 4.482 a	18.295 ± 0.422 a	1.435 ± 0.036
BC (50)	10.545 ± 0.328 a	12.339 ± 0.173 a	1.844 ± 0.031 b	256.388 ± 11.276 a	17.604 ± 0.688 ab	1.431 ± 0.064
BC (60)	9.757 ± 0.097 b	12.192 ± 0.061 ab	1.881 ± 0.006 b	254.969 ± 6.349 a	17.798 ± 0.277 ab	1.456 ± 0.033
BC (100)	9.093 ± 0.076 c	11.803 ± 0.105 b	1.863 ± 0.015 b	244.138 ± 1.633 a	17.485 ± 0.261 ab	1.496 ± 0.05

Note: Root tips (RT), root forks (RF), root crossing (RC), specific root length (SRL), specific root area (SRA), root tissue density (RTD). Values are shown as mean ± standard error (n = 3). The significance of differences was determined by one-way analysis of variance (ANOVA) using Duncan’s multiple range test. Different letters indicate significant differences at *p* ≤ 0.05.

**Table 5:** Evaluation of physiological characteristics of plant growth under different treatments.

Index	Treatment						
	BC (0)	BC (20)	BC (30)	BC (40)	BC (50)	BC (60)	BC (100)
PHInc	0.131	0.606	0.749	0.990	0.701	0.569	0.374
GDInc	0.483	0.804	0.859	0.575	0.439	0.156	0.121
LFW	0.068	0.575	0.690	0.893	0.914	0.655	0.204
RFW	0.146	0.554	0.676	0.725	0.613	0.429	0.034
SFW	0.024	0.385	0.629	0.558	0.616	0.318	0.396
TFW	0.010	0.485	0.639	0.721	0.666	0.420	0.044
LDW	0.305	0.504	0.569	0.762	0.841	0.487	0.202
RDW	0.146	0.383	0.487	0.699	0.542	0.155	0.048
SDW	0.054	0.452	0.659	0.642	0.699	0.409	0.578
TDW	0.082	0.380	0.511	0.687	0.643	0.240	0.128
SUG	0.137	0.860	0.485	0.696	0.562	0.330	0.247
PROT	0.187	0.612	0.678	0.431	0.307	0.258	0.031
Chla	0.462	0.759	0.815	0.678	0.437	0.157	0.061
Chlb	0.086	0.758	0.787	0.550	0.611	0.859	0.253
a/b	0.186	0.606	0.667	0.419	0.308	0.237	0.032
TRL	0.035	0.441	0.647	0.837	0.909	0.839	0.614
TPA	0.033	0.377	0.565	0.875	0.914	0.855	0.527
TSA	0.076	0.364	0.633	0.913	0.847	0.817	0.671
AD	0.523	0.487	0.277	0.267	0.347	0.275	0.232
RV	0.064	0.187	0.564	0.754	0.771	0.743	0.650
SRL	0.040	0.285	0.566	0.782	0.820	0.728	0.567
SPA	0.011	0.526	0.477	0.512	0.623	0.598	0.405
RTD	0.114	0.412	0.549	0.773	0.637	0.675	0.614
RT	0.744	0.500	0.530	0.462	0.450	0.524	0.643
RF	0.051	0.286	0.383	0.702	0.762	0.560	0.390
RC	0.030	0.361	0.496	0.756	0.870	0.833	0.735
Tr	0.010	0.485	0.639	0.721	0.666	0.420	0.044
Pn	0.305	0.504	0.569	0.762	0.841	0.487	0.202
Ci	0.146	0.383	0.487	0.699	0.542	0.155	0.048
Gsw	0.054	0.452	0.659	0.642	0.699	0.409	0.578
AS	0.158	0.486	0.601	0.677	0.656	0.535	0.367
Rank	7	5	3	1	2	4	6

Notes: AS, average score.

## Data Availability

All data generated or analyzed during this study are included in this published article (Supplementary Files) and also available from the corresponding author on reasonable request.

## Supplementary Materials

The supporting information can be downloaded at: https://www.mdpi.com/article/10.3390/plants13131736/s1.
